# Association of serum magnesium and calcium with metabolic syndrome: a cross-sectional study from the Qatar-biobank

**DOI:** 10.1186/s12986-024-00892-y

**Published:** 2025-01-30

**Authors:** Raneem Alsheikh, Haneen Aldulaimi, Rami Hinawi, Fatima Al-Sadi, Alanoud Al-Baker, Aldana Alkuwari, Muhammad Sameer, Ghalya Al-Abdulla, Zumin Shi, Giridhara Rathnaiah Babu

**Affiliations:** 1 College of Medicine, QU Health, Qatar University, P.O. Box 2713, Doha, Qatar; 2 Human Nutrition Department, College of Health Sciences, QU Health, Qatar University, P.O. Box 2713, Doha, Qatar; 3 Department of Population Medicine, College of Medicine, QU Health, Qatar University, P.O. Box 2713, Doha, Qatar

**Keywords:** Metabolic syndrome (MetS), Serum magnesium (Mg), Serum calcium (Ca), Calcium-to-magnesium ratio, Qatar biobank (QBB)

## Abstract

**Background and objectives:**

Metabolic syndrome (MetS) and its constituent comorbidities, along with mineral imbalances, pose a significant health burden in the Qatari population. Although Magnesium (Mg) and Calcium (Ca) have been individually linked to MetS, the impact of the calcium-to-magnesium ratio (Ca: Mg) on MetS remains unclear, especially in the adult population of Qatar. In this study, we aim to investigate the association between the total serum concentrations of Ca, Mg and Ca: Mg ratio with the outcome of MetS.

**Methods:**

This comprehensive cross-sectional study included data on 9693 participants collected by Qatar Biobank (QBB). The serum levels of Mg and Ca, in addition to recorded metabolic parameters for the study participants, were used in the analyses. The presence of MetS was deemed as our primary outcome and its components as secondary outcomes. Logistic regression models were run to examine these associations.

**Results and conclusion:**

MetS was present in more than 19% of the population. The mean serum Mg was higher in the non-MetS group 0.83 ± 0.06 mmol/L compared to the MetS group 0.81 ± 0.08 mmol/L. Conversely, the mean serum Ca and Ca: Mg ratio were higher in the MetS group (2.33 ± 0.09 mmol/L, 2.92 ± 0.36 mmol/L) compared to the non-MetS group (2.30 ± 0.08 mmol/L, 2.77 ± 0.23 mmol/L) respectively. In the context of MetS, we observed a negative dose-response relationship between Mg quartiles and MetS. In contrast, we found a positive association between Ca as well as Ca: Mg ratio and MetS.

**Supplementary Information:**

The online version contains supplementary material available at 10.1186/s12986-024-00892-y.

## Introduction

Metabolic Syndrome (MetS) is a cluster of cardiovascular risk factors, including hypertension, hyperglycemia, hypertriglyceridemia, low levels of high-density lipoprotein (HDL), and central obesity [1]. MetS is highly prevalent worldwide, with Qatar exhibiting a high prevalence, ranging from 26 to 33% [2]. This condition is driven by multiple risk factors, primarily a sedentary lifestyle, poor dietary habits, such as high intake of processed and fast foods, obesity, aging, and genetic predisposition [3, 4]. MetS has been significantly linked to serious complications, including type 2 diabetes, cardiovascular disease, and stroke [1]. Considering the pivotal burden of MetS, recent research has begun to underscore the potential role of micronutrients in the pathogenesis of this chronic condition [5–9].

Among these micronutrients, inorganic ions such as calcium (Ca) and magnesium (Mg) have attracted scientific attention. These ions are essential for sustaining various physiological activities in the human body, with Ca being primarily obtained from dairy products and Mg from whole grains and leafy greens [10, 11]. Mg is an important cofactor for more than 300 enzymes involved in major body functions [12], including regulating glucose metabolism [13], heart rhythm [14], blood pressure [15], platelet-dependent thrombosis [16], and neurological functions [13]. On the other hand, Ca plays a crucial role in cardiac and skeletal muscle contractility [17, 18], senescence [19], cellular signaling [20], and blood pressure regulation [21].

Subclinical Mg deficiency is a common global issue, affecting between 10 and 30% of people in developed countries [22]. Yet, it is often undetected, as serum Mg is not routinely measured in most patients. In Qatar, micronutrients deficiency is primarily influenced by the lifestyle factors and dietary patterns of the population, such as excessive consumption rates of refined grains [23], fast food, and desalinated water [24]. While global data on Ca deficiency, including in Qatar remains limited, existing literature suggests that Ca and vitamin D supplementation may reduce the risk of cardiovascular diseases [25], highlighting the importance of examining Ca levels in relation to MetS in this population.

Evidence indicates that Ca and Mg are intricately related, acting as key antagonists to each other [26]. Considering this, examining the Ca: Mg ratio provides valuable insights into the complex interplay between these two essential micronutrients, providing deeper insights on their combined effects on health. Although some studies have investigated the individual associations of Ca and Mg with MetS [15, 27–29], the collective impact of their ratio (Ca: Mg) on metabolic health has not been thoroughly explored yet. In this study, we aim to examine the associations of Ca, Mg, and Ca: Mg with MetS using data from the Qatar-biobank (QBB).

## Methods

### Study design and sample

QBB is a prospective population-based study that was established in 2012. It recruited adult participants who were Qatari nationals or residents of Qatar for more than 15 years. The QBB study collected information on sociodemographic factors, lifestyle, and dietary habits through self-administered questionnaires. Nurse interviews were conducted to collect information on self- and family history of health conditions and medications use. All participants underwent a health examination in the QBB facility at Hamad Medical Center [30].

In our cross-sectional study, we included a random sample of 9693 participants aged 20 years and above, with complete serum Mg, Ca, and metabolic parameters data. Those with missing data on serum Mg, Ca, metabolic parameters or an estimated glomerular filtration rate (eGFR) of less than 60 mL/min/1.73 m^2^ were excluded [31]. A total of 9653 participants entered the analyses. An informed consent was voluntarily obtained from all study participants. This study was approved by the QBB Institutional Review Board (IRB) (Ex-2024-QF-QBB-RES-ACC-00236-0290) as well as the Qatar University IRB (QU-IRB 193/2024-EM).

### Outcome variable: metabolic syndrome

MetS was defined, according to the harmonized definition in the literature, by the presence of three or more of the following components: (1) elevated waist circumference (≥ 102 cm in men, ≥ 88 cm in women); (2) elevated blood pressure (systolic ≥ 130 and/or diastolic ≥ 85 mm Hg) or taking an antihypertensive drug treatment; (3) reduced HDL cholesterol (< 1.0 mmol/L in men and < 1.3 mmol/L in women) or taking a drug treatment for low HDL; (4) elevated triglycerides (≥ 1.7 mmol/L) or taking a drug treatment for high TG; (5) elevated fasting glucose (≥ 5.6 mmol/L) or taking a drug treatment for elevated glucose [1]. Blood pressure was calculated as the average of three provided readings. All serum biomarkers were measured in the laboratories of Hamad Medical Centre Laboratory, Doha.

### Exposure variables: serum magnesium, calcium, and calcium-to-magnesium ratio

Serum Mg was measured by an automated colorimetric method (Magnesium Gen.2 from Roche Diagnostics, Indianapolis, IN) conducted in the central laboratory of QBB. The assay’s coefficients of variations are 0.3–0.8%. Subclinical magnesium deficiency was defined as serum Mg levels below 0.85 mmol/L, while hypocalcemia was defined as serum Ca levels below 2.1 mmol/L and hypercalcemia as serum Ca levels above 2.60 mmol/L [32]. Serum Ca was measured and corrected for serum albumin levels using the following equation: Ca (mmol/L) = Measured Ca (mmol/L) + 0.020 × (40 - Albumin (g/L) [33]. The Ca: Mg ratio was calculated by dividing the corrected serum concentration of Ca (mmol/L) by the serum concentration of Mg (mmol/L).

### Covariates

Based on prior evidence in the literature and determined through directed acyclic graphs (DAGs) (S1-S2), socioeconomic status and lifestyle factors were included in the analyses as covariates. They included age (separated by the median age into older and younger age groups), sex (male/female), nationality (Qatari or non-Qatari), smoking status (current smoker, non-smoker, ex-smoker), leisure time physical activity level (metabolic equivalent of task, reported as tertiles), and obesity (non-obese: BMI < 30 Kg/m2 and obese: BMI ≥ 30 kg/m2). Dyslipidemia was defined as having any of the following: total cholesterol ≥ 5.2 mmol/L, triglyceride ≥ 1.7 mmol/L, LDL cholesterol ≥ 3.4 mmol/L, or HDL cholesterol < 1.0 mmol/L in males and < 1.3 mmol/L in females [34]. Hyperglycemia was defined as either having elevated serum fasting glucose levels (≥ 5.6 mmol/L) or taking drug treatment for elevated glucose [1]. Vitamin D status was classified into three groups based on serum vitamin D concentrations: adequacy as ≥ 20 ng/ml, inadequacy as < 20 ng/ml and ≥ 12 ng/ml, and deficiency as < 12 ng/ml [35]. eGFR was calculated using the CKD-EPI creatinine Eq. [31].

A food frequency questionnaire (FFQ) was used to assess habitual food frequency intake, based on the FFQ used in the European Prospective Investigation of Cancer (EPIC) study. The FFQ included 102 foods items that are commonly consumed in Qatar. The 102 food items were collapsed into 38 groups based on similarity of the nutrient profiles and cooking methods (S3). Three dietary patterns were constructed using factor analysis: traditional, prudent, and sweets/fast food, similar to a previously conducted study in Qatar [36]. Factor loadings were presented in supplementary (S4). Dietary patterns were considered potential confounders in some of the analyses.

### Statistical analysis

The characteristics of the study sample were reported as means (SD) for normally distributed continuous variables, medians (IQR) for abnormally distributed continuous variables, and percentages for categorical variables. Participants with missing data were excluded (Section “Study design and sample”). Serum Ca, Mg, and Ca: Mg ratio concentrations were divided into quartiles. Three multivariable logistic regression models were run to assess the associations of serum Ca, Mg, and Ca: Mg ratio with MetS. All models were adjusted for covariates determined through DAGs (S1-S2, supplementary). The models assessing the association of serum Mg and Ca: Mg ratio with MetS were adjusted for age, sex, smoking status, physical activity, diet, and education. Similarly, the model examining serum Ca and MetS was adjusted for the same covariates with the addition of nationality. Secondary logistic regression analyses were conducted to determine the association between each component of MetS with serum Ca, Mg, and Ca: Mg ratio, separately. *Epitable3 command* was used to examine the multiplicative interactions between the primary outcome models and each of the following variables: age, sex, education, smoking status, physical activity, vitamin D status, supplement use, sleep duration, and PHQ-9. All analyses were run using Stata (Version 18, StataCorp, College Station, TX, USA). P-values and 95% confidence intervals (CIs) were reported as measures of compatibility with the data, as appropriate.

## Results

### Sample description

**Fig. 1:**
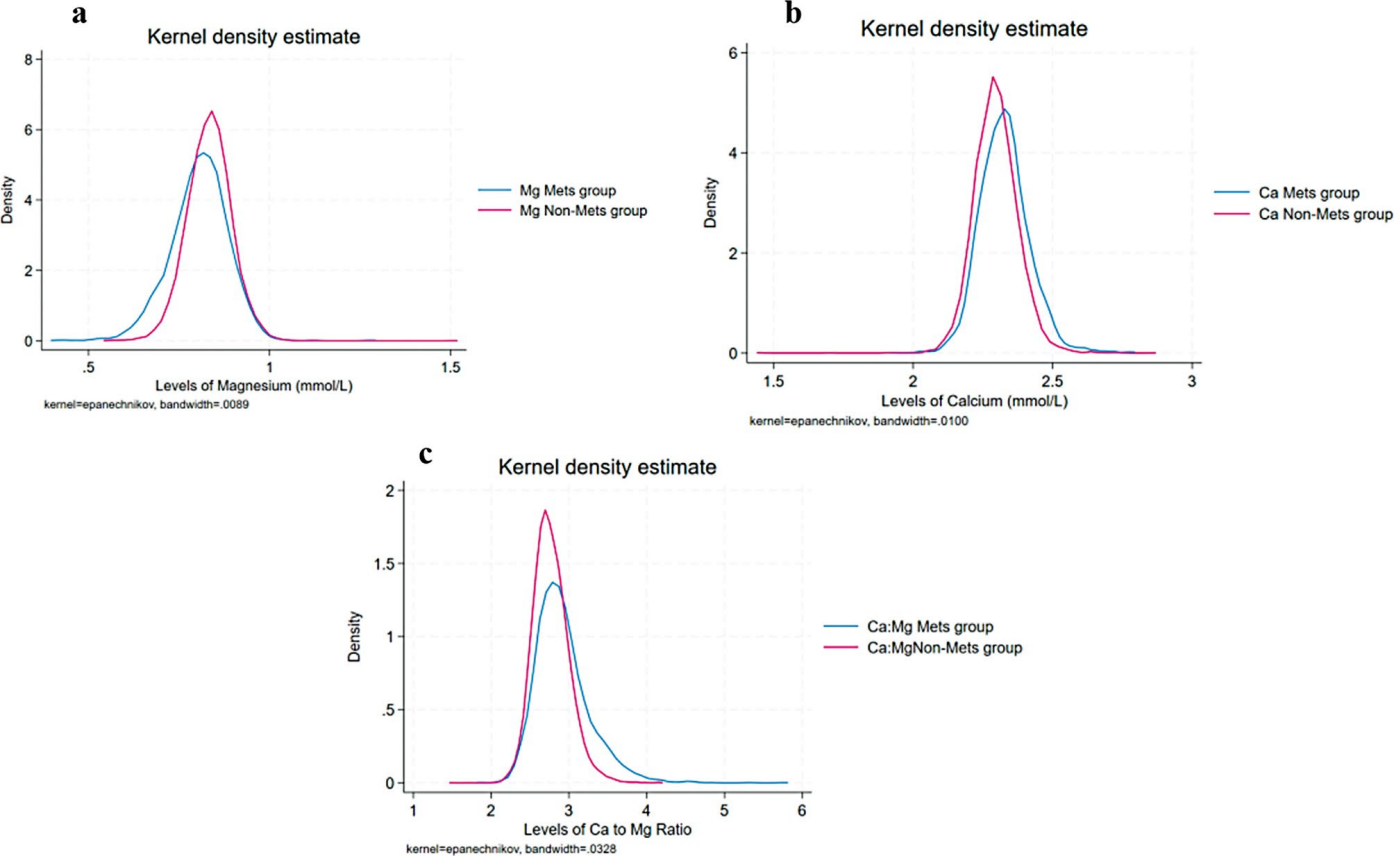
Distributions of Mg, Ca and Ca: Mg in serum by MetS status. Blue lines represent elemental levels in the MetS group, and red lines represent elemental levels in the non-MetS group. **a.** Distribution of Mg between MetS and non-MetS groups; **b**. Distribution of Ca between MetS and non-MetS groups; **c.** Distribution of Ca: Mg between MetS and non-MetS groups. Abbreviations: Ca, calcium; Mg, magnesium; Ca: Mg, calcium-to-magnesium ratio; Mets, metabolic syndrome group; Non-Mets, non-metabolic syndrome group

The average age of the total study population (9653) was 39.9 ± 11.4 years. Overall, most of the study participants were Qataris (87%), highly educated (55.2%), and non-smokers (64%). Notably, the leisure-time physical activity was low among all participants. The characteristics of the total study sample, by MetS status, were summarized in Table 1, further stratification by Ca and Mg quartiles was done and presented in supplementary (S5-S6). MetS was present in more than 19% of the study population, affecting 55.7% of women and 44.3% of men in the study. The mean serum Mg was higher in the non-MetS group 0.83 ± 0.06 mmol/L compared to the MetS group at 0.81 ± 0.08 mmol/L (Fig. 1a). In contrast, the mean serum Ca was higher in the MetS group 2.33 ± 0.09 mmol/L compared to the non-MetS group at 2.30 ± 0.08 mmol/L(Fig. 1b). MetS group was mainly distributed in a high Ca: Mg area, while non-MetS in a low Ca: Mg area (Fig. 1c). Subjects in the MetS group had higher triglycerides, systolic blood pressure (SBP), diastolic blood pressure (DBP), fasting plasma glucose, and waist circumference as well as lower total cholesterol, HDL, LDL, eGFR, and leisure time physical activity levels than subjects in the non-MetS group.

**Table 1:** Sample characteristics by metabolic syndrome status (MetS) among participants attending Qatar biobank study (*N* = 9653)

	Non-MetS group	MetS group	*p*-value
	*N* = 7,724	*N* = 1,929	
Age (Years)	36.00 (29.00–44.00)	52.00 (45.00–58.00)	< 0.001
Sex			0.032
Male Female	3,630 (47.0%)	854 (44.3%)	
	4,094 (53.0%)	1,075 (55.7%)	
Education			< 0.001
Low Medium High	910 (11.8%)	673 (34.9%)	
	2,344 (30.4%)	394 (20.4%)	
	4,468 (57.9%)	862 (44.7%)	
Smoking Status			< 0.001
Non-Smoker Smoker Ex-Smoker	4,702 (62.6%)	1,321 (70.2%)	
	1,529 (20.4%)	297 (15.8%)	
	1,277 (17.0%)	265 (14.1%)	
Nationality			0.028
Non-Qatari Qatari	976 (12.6%)	280 (14.5%)	
	6,748 (87.4%)	1,649 (85.5%)	
Income			< 0.001
<20k 20k-50k >80k	2,722 (38.2%)	783 (44.2%)	
	2,733 (38.4%)	531 (30.0%)	
	1,671 (23.4%)	456 (25.8%)	
Leisure time physical activity (hours/week)	6.00 (0.00–27.00)	0.00 (0.00–12.00)	< 0.001
Sleep Duration			< 0.001
≥7 h <7 h	2,984 (38.7%)	665 (34.5%)	
	4,736 (61.3%)	1,264 (65.5%)	
Obesity			< 0.001
No Yes	4,854 (62.9%)	504 (26.1%)	
	2,869 (37.1%)	1,424 (73.9%)	
eGFR (mL/min/1.73 m2)	142.1 (13.0)	139.1 (17.4)	< 0.001
Supplements Use			< 0.001
No Yes	3,247 (42.0%)	688 (35.7%)	
	4,477 (58.0%)	1,241 (64.3%)	
Vitamin D (ng/ml)	15.0 (11.0–22.0)	20.0 (14.0–26.0)	< 0.001
Dietary Patterns			
Traditional Prudent Sweets/Fast Food	0.08 (1.02)	-0.32 (0.81)	< 0.001
	-0.04 (1.02)	0.16 (0.89)	< 0.001
	0.02 (1.01)	-0.09 (0.96)	< 0.001
Lipid Parameters			
Cholesterol Total (mmol/L)	5.0 (0.9)	4.9 (1.0)	< 0.001
HDL Cholesterol Total (mmol/L)	1.4 (0.3)	1.2 (0.3)	< 0.001
Triglyceride (mmol/L)	1.0 (0.8–1.4)	1.6 (1.1–2.1)	< 0.001
LDL Cholesterol Total (mmol/L)	3.0 (0.8)	2.9 (0.9)	< 0.001
Glucose (mmol/L)	4.9 (4.5–5.3)	6.2 (5.6–8.5)	< 0.001
Blood Pressure			
Systolic Blood Pressure (mmHg) Diastolic Blood Pressure (mmHg)	111 (12)	127 (15)	< 0.001
	66 (9)	71(10)	< 0.001
Waist Circumference	86.6 (13.1)	101.4 (11.7)	< 0.001
Magnesium (mmol/L)	0.83 (0.06)	0.81 (0.08)	< 0.001
Quartiles of Magnesium			< 0.001
Q1 (0.41–0.79)	1,980 (25.6%)	807 (41.8%)	
Q2 (0.80–0.83)	1,906 (24.7%)	419 (21.7%)	
Q3 (0.84–0.87)	2,006 (26.0%)	367 (19.0%)	
Q4 (0.88–1.51)	1,832 (23.7%)	336 (17.4%)	
Calcium (mmol/L)	2.30 (0.08)	2.33 (0.09)	< 0.001
Quartiles of Calcium			< 0.001
Q1 (1.45–2.25)	2,282 (29.5%)	379 (19.6%)	
Q2 (2.26–2.30)	2,051 (26.6%)	418 (21.7%)	
Q3 (2.31–2.35)	1,767 (22.9%)	468 (24.3%)	
Q4 (2.36–2.86)	1,624 (21.0%)	664 (34.4%)	
Calcium-to-Magnesium Ratio	2.77 (0.23)	2.92 (0.36)	< 0.001
Quartiles of Calcium-to-Magnesium Ratio			< 0.001
Q1 (1.49–2.62)	2,094 (27.1%)	335 (17.4%)	
Q2 (2.62–2.77)	2,038 (26.4%)	364 (18.9%)	
Q3 (2.77–2.93)	1,978 (25.6%)	445 (23.1%)	
Q4 (2.93–5.75)	1,614 (20.9%)	785 (40.7%)	

Abbreviations: MetS, metabolic syndrome group; Non-MetS, non-metabolic syndrome group; LDL, low-density lipoproteins; HDL, high-density lipoproteins; eGFR, estimated glomerular filtration rate

### Association between serum magnesium, calcium, and calcium-to-magnesium ratio quartiles and metabolic syndrome

Table 2 lists the results of the multivariable logistic regression analyses for MetS and the quartiles of serum Ca, Mg, and Ca: Mg ratio. The results showed that lower serum Mg levels were associated with higher odds of MetS suggesting a negative-dose-response relationship (P for Trend < 0.001). Moving from Q1 to Q3 of Mg, there was 2.41 (95% CI 2.05–2.82), 1.41 (95% CI 1.18–1.68), and 1.11 (95% CI 0.93–1.32) fold increase in the odds of MetS, compared to Q4, respectively. Across Ca quartiles, Ca showed a positive association with MetS. Remarkably, Q4 (2.36–2.86) of Ca demonstrated a 1.73 (95% CI 1.47–2.02) fold increase in the odds of MetS. Similarly, moving from Q1 to Q3, the Ca: Mg ratio depicted a positive dose-response relationship with MetS, represented by an incline in the odds ratios across its quartiles: 1.17 (95% CI 0.99–1.40), 1.58 (95% CI 1.33–1.87), and 2.74 (95% CI 2.33–3.22), respectively.

**Table 2:** Association between serum magnesium, calcium, and calcium-to-magnesium ratio quartiles and metabolic syndrome

Outcome	Quartiles	Odds Ratio	*P*-Value	95% CI	*P* for Trend
Metabolic Syndrome					< 0.001
	**Magnesium** ^**1**^				
	Q1	2.41	< 0.001	(2.05–2.82)	
	Q2	1.41	< 0.001	(1.18–1.68)	
	Q3	1.11	0.228	(0.93–1.32)	
	Q4	1.00			
	**Calcium** ^**2**^				
	Q1	1.00			< 0.001
	Q2	1.17	0.059	(0.99–1.38)	
	Q3	1.40	< 0.001	(1.19–1.66)	
	Q4	1.73	< 0.001	(1.47–2.02)	
	**Calcium-to-Magnesium Ratio** ^**3**^				
	Q1	1.00			< 0.001
	Q2	1.17	0.064	(0.99–1.40)	
	Q3	1.58	< 0.001	(1.33–1.87)	
	Q4	2.74	< 0.001	(2.33–3.22)	

^1^Model was adjusted for age, sex, smoking status, physical activity, diet, education (*n* = 9389). ^2^Model was adjusted for age, sex, smoking status, physical activity, diet, education, and nationality (*n* = 9389). ^3^Model was adjusted for age, sex, diet, smoking status, physical activity, education (*n* = 9389. Abbreviations: CI, Confidence Interval; Q1, Quartile 1; Q2, Quartile2; Q3, Quartile 3; Q4, Quartile 4

### Association between serum magnesium, calcium, and calcium-to-magnesium ratio quartiles and metabolic syndrome components

Individual secondary analyses between serum Mg, Ca, and Ca: Mg and each component of MetS were conducted (S7-S8, Supplementary). Although our analyses revealed that lower serum Mg levels were consistently associated with higher odds of elevated triglycerides, reduced HDL, elevated glucose, and central obesity, elevated glucose had the most prominent association with low Mg levels. We also found that elevated serum Ca levels were positively associated with elevated triglycerides, glucose, and blood pressure levels. Notably, the associations with elevated blood pressure and triglycerides stood out, with a 1.71 and 1.72-fold increase in odds at Q4, respectively. The collective association of both micronutrients is presented in Table 3 through the Ca: Mg ratio. All components of MetS demonstrated a positive dose response relationship with Ca: Mg quartiles (p for trend < 0.001). Among the five components, elevated glucose stood out with a 170% increase in odds at Q4 (95% CI 2.35–3.12).

**Table 3:** Association between serum calcium-to-magnesium ratio (ca: mg) and components of metabolic syndrome

Outcome	Calcium-to-magnesium ratio quartiles	Odds ratio	*P*-value	95% CI	*P* for trend
Elevated Triglycerides^1^					
	Q1	1.00			< 0.001
	Q2	1.08	0.421	(0.88–1.34)	
	Q3	1.22	0.048	(1.00-1.50)	
	Q4	2.03	< 0.001	(1.68–2.44)	
Reduced HDL^2^					
	Q1	1.00			< 0.001
	Q2	1.05	0.438	(0.92–1.19)	
	Q3	1.10	0.129	(0.97–1.24)	
	Q4	1.57	< 0.001	(1.39–1.78)	
Elevated glucose^3^					
	Q1	1.00			< 0.001
	Q2	1.17	0.037	(1.00-1.36)	
	Q3	1.48	< 0.001	(1.28–1.72)	
	Q4	2.70	< 0.001	(2.35–3.12)	
Elevated blood pressure^4^					
	Q1	1.00			< 0.001
	Q2	0.99	0.927	(0.84–1.16)	
	Q3	1.16	0.059	(0.99–1.37)	
	Q4	1.64	< 0.001	(1.41–1.91)	
Central obesity^5^					
	Q1	1.00			< 0.001
	Q2	1.08	0.259	(0.94–1.23)	
	Q3	1.23	0.002	(1.08–1.41)	
	Q4	1.53	< 0.001	(1.34–1.75)	

^1^Model was adjusted for age, obesity, smoking status, physical activity, vitamin D status (*n* = 9334). ^2^Model was adjusted for age, sex, obesity, smoking status (*n* = 9393). ^3^Model was adjusted for age, obesity, smoking status, physical activity (*n* = 9398). ^4^Model was adjusted for age, obesity, smoking status, physical activity, dyslipidemia, diet (*n* = 9395). ^5^Model was adjusted for age, sex, smoking status, physical activity, vitamin D status, diet, elevated glucose (*n* = 9336). Abbreviations: HDL, high-density lipoproteins; CI, confidence interval; Q1, Quartile 1; Q2, Quartile2; Q3, Quartile 3; Q4, Quartile 4

### Subgroup analyses

In subgroup analyses (S9-S11, supplementary), we found no significant interaction between serum Mg and Ca: Mg ratio with any of the following variables: age, sex, education, smoking status, physical activity, vitamin D status, supplement use, sleep duration, and PHQ-9. However, sex and smoking status exhibited significant interactions with the serum Ca and MetS model (p for interaction 0.004 and 0.029, respectively). As for sex, at Q4, females had a 2.24-fold increase in the odds of MetS (95% CI 1.79–2.81) compared to males with OR of 1.29 (95% CI 1.03–1.62).  Moving to smoking status, non-smokers displayed a 2.06-fold increase in the odds of MetS (95% CI 1.70-2.51) compared to smokers with odds of 1.22 (95% CI 0.83-1.79), at Q4.

## Discussion

In this large cross-sectional study, we investigated the association of serum Ca, Mg, and Ca: Mg ratio with MetS. Our findings showed a consistent inverse or protective dose-response relationship between Mg levels and MetS, this association remained consistent with most components of MetS. In contrast, serum Ca and Ca: Mg ratio levels demonstrated a positive association with MetS across all quartiles. Among all components of MetS, elevated blood pressure and triglycerides had the most prominent associations with Ca, whereas elevated glucose showed the strongest association with Mg as well as the Ca: Mg ratio.

### Magnesium and metabolic syndrome

Our findings conveyed that Mg may have a protective effect, as evidenced by its inverse relationship with MetS across a negative gradient defined by its quartiles. This association became particularly notable in relation to elevated glucose among the MetS components. The inverse association between Mg and MetS could be explained by the role Mg plays in modulating diverse biochemical pathways that drive certain components of MetS, such as insulin resistance and dyslipidemia. For instance, evidence showed that as intracellular Mg levels fall, insulin receptor tyrosine kinase phosphorylation declines, leading to reduced uptake of glucose into the cells, thus insulin resistance [37]. Additionally, it has been established that adequate Mg levels are required for optimal metabolic activity, as Mg ions regulate major triglyceride lowering and glycolysis rate-limiting enzymes. This explains how low Mg levels can disrupt lipid metabolism, leading to dyslipidemia [38, 39].

Our findings align with several studies that have consistently reported a significant correlation between low Mg levels and MetS [40–42]. A meta-analysis of 5496 participants found a significant difference in Mg levels between MetS cases and controls (SMD = − 0.98, 95% CI = − 1.44 to − 0.52), suggesting an inverse association between Mg levels and MetS [43]. Similarly, findings from the 15-year CARDIA study showed a marked reduction in the risk of MetS among participants at the top two quartiles of Mg consumption. Those at the highest quartile had a 31% lower risk (HR, 0.69; 95% CI, 0.52 to 0.91; P for trend < 0.01) compared to those at the lowest quartile [44]. However, some studies found contrasting results. A Chinese case-control study reported higher median Mg levels in the MetS group (34.30 mg/l) compared to controls (32.32 mg/l) [15]. Furthermore, a study conducted in Korea found no significant difference in Mg levels between MetS cases and controls [45]. This inconsistency could be attributed to differences in the dietary patterns among Chinese, Korean, and Qatari populations. To elaborate, Chinese diets typically include more Mg-rich foods like dark green vegetables, whole grains, and beans [46]. Similarly, Korean diets, which often feature fermented vegetables, rice, and seafood, also provide significant amounts of Mg [47]. In contrast, the Qatari diet is characterized by a higher consumption of refined grains, fast food, and desalinated water, which are low in Mg [23, 48]. Considering that the studies in Chinese and Korean populations did not adjust for dietary factors in their models, while our analysis accounted for these variations, could help explain the discrepancies observed between our findings.

### Calcium and metabolic syndrome

Our findings showed a positive association between serum Ca and MetS, which became particularly pronounced with elevated blood pressure and triglycerides. This association can be clarified by exploring distinct mechanisms through which Ca modulates blood pressure regulation and dyslipidemia, thereby contributing to the complex pathophysiology underlying MetS. To elaborate, Ca is well-recognized for its distinct role in the regulation of muscle contraction. However, an imbalance in Ca levels can disrupt its physiological functions, particularly when levels are abnormally elevated. Such dysregulation may contribute to the pathophysiology of hypertension by excessively inducing spasms in vascular smooth muscle, thereby increasing peripheral resistance, promoting platelet aggregation, and elevating blood viscosity [49]. It has also been shown that Ca is associated with dyslipidemia [50, 51]. Evidence indicates that high Ca levels can disrupt lipids catabolism in the liver by increasing lipid synthesis and decreasing its absorption leading to higher lipid levels in the blood [52].

Our results align with most findings in the literature [28, 53, 54]. A cross-sectional study conducted by Chen et al. elucidated a significant association between MetS and elevated Ca levels (OR = 2.28, 95% CI: 1.42–3.69), as well as its constituent components, including elevated blood pressure, glucose, and triglycerides [55]. Additionally, research on middle-aged Korean men demonstrated that serum Ca levels were positively correlated with MetS risk score (*r* = 0.1769, *p* < 0.01) [28]. On the other hand, a few studies reported an inverse association between Ca intake and MetS [56–58]. For instance, a meta-analysis involving 63,017 participants indicated that higher dietary Ca intake was significantly associated with a decreased incidence of MetS (RR: 0.89; 95% CI: 0.80–0.99; I2 = 75.3) [58]. Findings from the existing literature revealed inconsistent results regarding the relationship between serum Ca levels and MetS, as well as that of dietary Ca intake and MetS. Therefore, further research is warranted to untangle this discrepancy and clarify the underlying mechanisms.

### Calcium-to-magnesium ratio and metabolic syndrome

Ca: Mg ratio has garnered a significant scientific attention in recent years and has been associated with diverse health outcomes, such as mortality from coronary artery disease [59], postmenopausal breast cancer [60], and psychiatric disorders [61]. However, considering that the Ca: Mg ratio remains an evolving concept in literature, there are relatively few studies examining its implications in relation to metabolic function [15]. Our findings contribute novel insights by indicating that a high Ca: Mg ratio is positively associated with MetS in Qatar, serving as a stronger predictor of MetS than Ca or Mg levels when assessed individually. This is validated by evidence showing that Ca and Mg act as natural antagonists, where an imbalance in one can lead to a disruption in the other [62].

Ca and Mg act as competing antagonists, both targeting the same receptors for binding at the cellular level. To elaborate, when Mg levels drop significantly, increased Ca transport into the cells occurs, leading to secondary hypocalcemia [63]. Conversely, when Ca levels become excessively elevated, they inhibit Mg reabsorption in the kidneys by activating the calcium-sensing receptor (CaSR) in the thick ascending limb. This activates a signaling cascade where the expression of Claudin-14 disrupts Mg reabsorption, contributing to hypermagnesuria and hypercalciuria [64, 65], which can further exacerbate existing subclinical hypomagnesemia. The complex interaction between Ca and Mg at the cellular level necessitates a delicate balance to enhance their effects, and merely having each mineral within its respective normal range fails to capture this dynamic relationship. This was demonstrated in a prospective study done on hospitalized patients, which found that administering intravenous infusions of Mg to normomagnesemic hypocalcemic patients helped normalize their serum Ca levels [66]. Ultimately, given the cost-effectiveness and accessibility of serum Ca and Mg measurements in most clinical laboratories along with the Ca: Mg ratio’s value in capturing their combined homeostatic impact, we advocate for incorporating Ca: Mg ratio monitoring into routine clinical practice to support tailored interventions for high-risk individuals. Further research is essential to validate and expand upon these findings.

### Strengths and limitations

To the best of our knowledge, this is the largest study in literature and the first in Qatar and the Middle East to evaluate the relationship between serum Ca, Mg, and Ca: Mg levels with MetS. In addition, using DAGs, we were able to account for important confounders in our models, ensuring robust and reliable analyses. While our study offers valuable insights, it is essential to acknowledge limitations inherent to its cross-sectional design, which preclude any inference of causality or temporality between Ca, Mg, and MetS. Additionally, the lack of information on some key variables,  such as parathyroid hormone (PTH) levels, history of antacid and diuretic use, alcohol consumption, and menopausal status in females—further limits our study. Moreover, residual confounding due to both known and unknown covariates, represents another limitation in this study.

## Conclusion

To conclude, our primary outcome analyses suggested an inverse association between serum Mg and MetS; conversely, they indicated a positive association between Ca and Ca: Mg ratio and MetS. A high Ca: Mg ratio showed a stronger association with MetS than the individual impact of Ca or Mg alone. Further longitudinal studies are warranted to establish the temporal nature of these associations and develop targeted management options for metabolic dysfunctions.

## Electronic supplementary material

Below is the link to the electronic supplementary material.

Supplementary Material 1

## Data Availability

The data for this study was obtained from the Qatar BioBank (QBB). Due to QBB’s restrictions on data availability, this data was used under license for this study.

## Abbreviations

MetS: Metabolic Syndrome
Ca: Calcium
Mg: Magnesium
Ca: Mg: Calcium-to-magnesium ratio
QBB: Qatar BioBank
BMI: Body Mass Index
DAGs: Directed Acyclic Graphs
LDL: Low-Density Lipoproteins
HDL: High-Density Lipoproteins
eGFR: Estimated Glomerular Filtration Rate
FFQ: Food Frequency Questionnaire
SBP: Systolic Blood Pressure
DBP: Diastolic Blood Pressure
LCAT: Lecithin Cholesterol Acyltransferase
LPL: Lipoprotein Lipase
PTH: Parathyroid Hormone
