# Supplementary material for: Association of serum magnesium and calcium with metabolic syndrome: a cross-sectional study from the Qatar-biobank
Source: Nutr Metab (Lond). 2025 Jan 30;22:8. doi: 10.1186/s12986-024-00892-y (PMC11783880; doi:10.1186/s12986-024-00892-y)
Supplement: Supplementary file 1 — Supplementary Material 1 [file 12986_2024_892_MOESM1_ESM.docx]

***Supplementary Material***

**S1** Magnesium and Metabolic Syndrome DAG Diagram


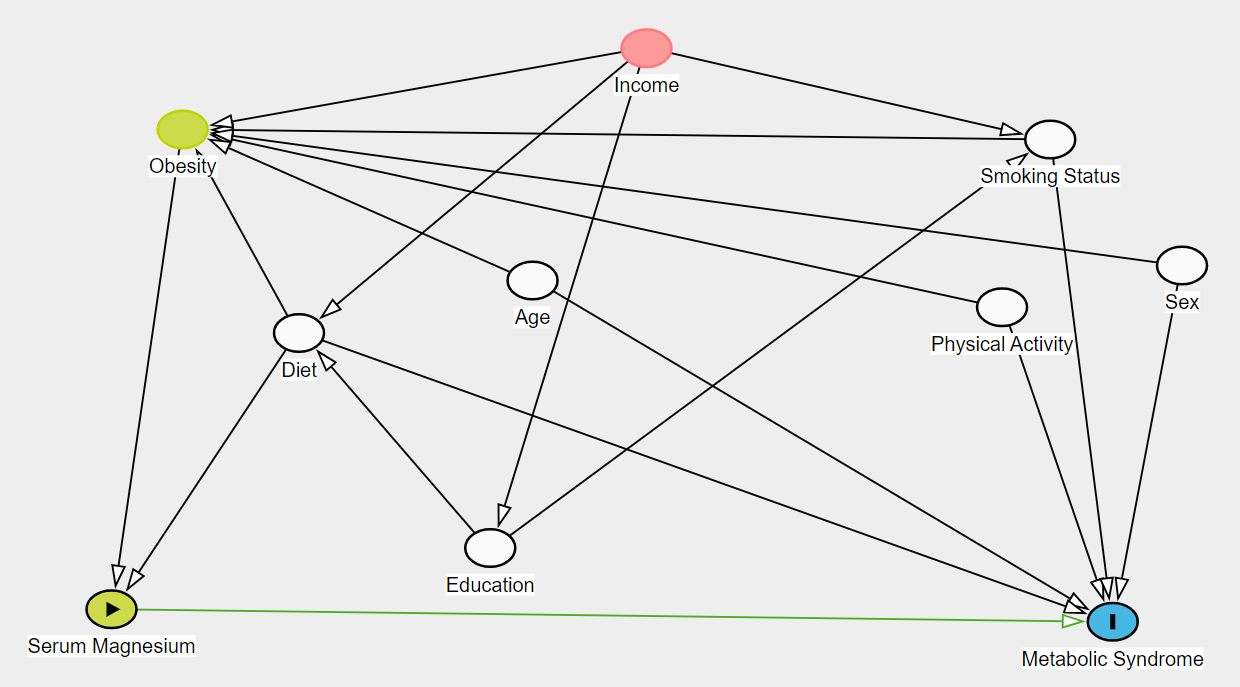


**S2** Calcium and Metabolic Syndrome DAG Diagram


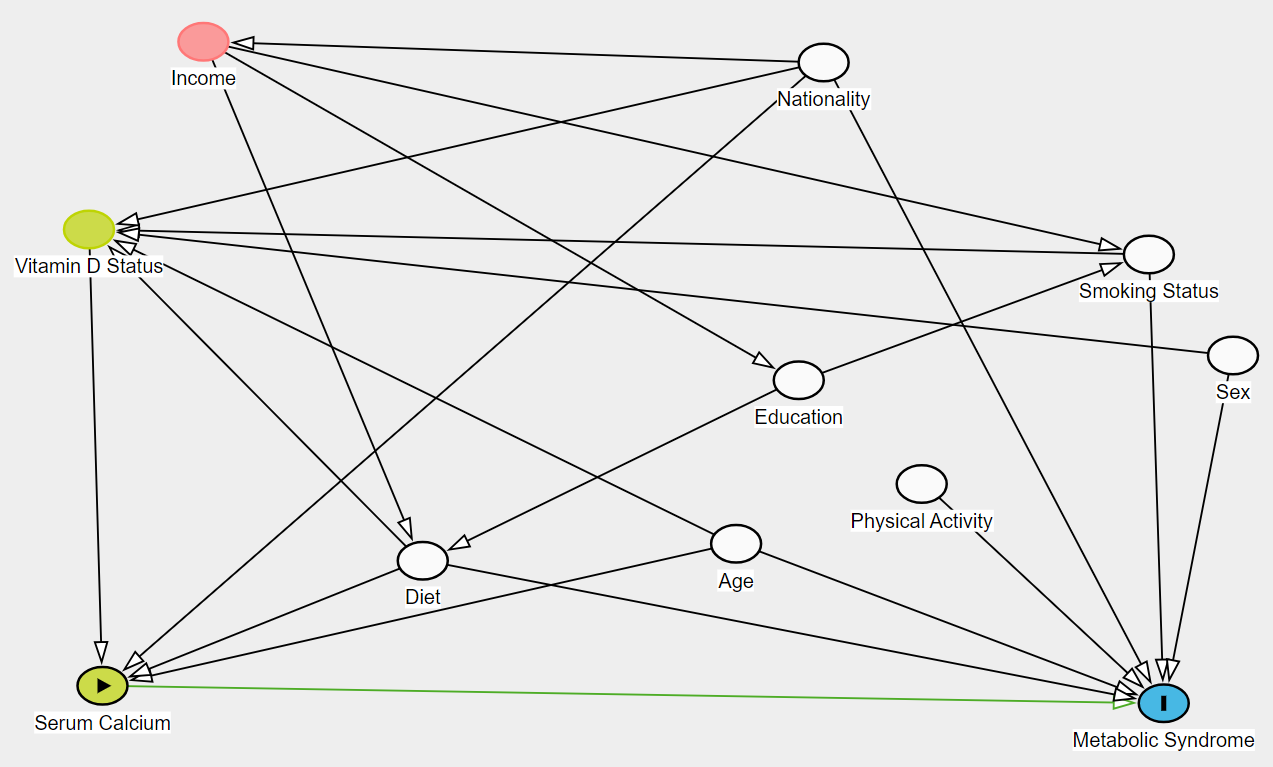


**S3** Food Groups Used in Factor Analysis

| Food group | Food items included |
| --- | --- |
| Coffee | Arabic coffee, instant coffee, other coffee (e.g. filter coffee, cappuccino) |
| Tea | Tea, herbal tea, Karak |
| Milk | Milk as a beverage (cold milk, cappuccino) |
| Milk added to cereal | Milk added to cereal |
| Milk shakes | Milk shakes or floured milk |
| Yoghurt | Laban, yoghurt, labneh |
| Cheese | Other white cheese (halloumi, cream cheese), processed cheese, hard cheese (such as gouda, cheddar) |
| Butter | Butter |
| Breakfast Cereal | Balaleet, cornflakes and other cold cereals, museli, porridge, other brand cereals |
| Arabic/Iranian bread | Arabic bread (lavash), Iranian bread |
| White bread | Toast (sliced bread, white) |
| Brown bread | Toast (sliced bread, brown) |
| Other bread | Other breads |
| Croissant | Croissant |
| Zaatar fatayer | Zaatar fatayer |
| Soups/starters | Ready-made soup from powder; other soups; hummus, mutabal, baba ghanush; other dips; tabbouleh; koussa mahshi (stuffed courgettes); eggplants fried or baked, moussaka; other vegetable starters; fish and seafoods starters; chicken, meat, or sausage starters |
| Salad and raw vegetables | Raw vegetables; Green leafy salad (e.g. lettuce); Mixed salad (for example with tomato, onion, cucumber, other vegetables); Fattoush; Beans salads (chickpeas, lentils); Bulghur salad |
| Salad and cooked vegetables | Vegetable stews; Vegetable curries; Other cooked vegetables; corn |
| Potato | Potato |
| White rice | White rice |
| Biryani | Biryani |
| Asian noodle | Asian noodle |
| Lasagna | Lasagna |
| Red meat | Red meat |
| Chicken | Chicken |
| Fish | Fish |
| Chicken/meat fish mixed dish | Meat served with rice (Mashboos); Chicken with rice; Harees; Meat cooked with vegetables (Margooga, Saloona); Chicken cooked with vegetables (Margooga, Saloona); Meat curry; Chicken curry; Kofta; Meat kebab; Chicken kebab; Lamb chops, escalope; Grilled chicken |
| Grilled/fried/baked Fish | Grilled, fried or baked fish; Fish cooked with vegetables; Smoked fish; |
| Eggs | Eggs (boiled, fried, omelettes, scrambled) |
| Fast food | Meat shawarma, Chicken shawarma, Falafel wrap, Samosa, Other middle eastern style fast foods, French fries, Potato chips, Burgers, hotdogs, Pizza, Fried chicken(e.g. wings, nuggets), |
| Fresh fruit | Fresh fruit, banana, watermelon |
| Canned/dried fruit and dates | Canned fruits, dry fruits, dates |
| Desserts | Traditional desserts (mahalabea, halwa, aquili, elgayemat, kammafaroursh); Other Middle Eastern or Lebanese desserts; Cookies, biscuits; Muffin, cake, doughnut |
| Chocolate | Chocolate |
| Ice cream | Ice cream |
| Nuts | Nuts |
| Fresh fruit juice | Fresh fruit juices; Smoothies |
| Soft drink | Preserved fruit juice (canned/bottled); Soft drinks, sodas; Diet soft drinks, sodas; Energy drinks |

**S4** Factor Loadings

**S5** Sample Characteristics by Magnesium Quartiles

|  | Q1 | Q2 | Q3 | Q4 | p-value |
| --- | --- | --- | --- | --- | --- |
|  | N=2,790 | N=2,328 | N=2,377 | N=2,169 |  |
| Magnesium (mmol/L) | 0.75 (0.04) | 0.82 (0.01) | 0.85 (0.01) | 0.91 (0.04) | <0.001 |
| Metabolic Syndrome |  |  |  |  | <0.001 |
| No | 1,980 (71.0%) | 1,906 (82.0%) | 2,006 (84.5%) | 1,832 (84.5%) |  |
| Yes | 807 (29.0%) | 419 (18.0%) | 367 (15.5%) | 336 (15.5%) |  |
| Calcium (mmol/L) | 2.31 (0.09) | 2.30 (0.08) | 2.30 (0.08) | 2.30 (0.08) | <0.001 |
| Quartiles of Calcium |  |  |  |  | <0.001 |
| Q1 | 671 (24.1%) | 654 (28.1%) | 704 (29.6%) | 634 (29.2%) |  |
| Q2 | 668 (23.9%) | 581 (25.0%) | 644 (27.1%) | 578 (26.6%) |  |
| Q3 | 670 (24.0%) | 553 (23.8%) | 525 (22.1%) | 491 (22.6%) |  |
| Q4 | 781 (28.0%) | 540 (23.2%) | 504 (21.2%) | 466 (21.5%) |  |
| Ca to Mg Ratio | 3.09 (0.25) | 2.82 (0.10) | 2.69 (0.10) | 2.52 (0.12) | <0.001 |
| Quartiles of Ca to Mg Ratio |  |  |  |  | <0.001 |
| Q1 | 6 ( 0.2%) | 54 ( 2.3%) | 609 (25.6%) | 1,762 (81.2%) |  |
| Q2 | 45 ( 1.6%) | 677 (29.1%) | 1,308 (55.0%) | 375 (17.3%) |  |
| Q3 | 656 (23.5%) | 1,315 (56.5%) | 426 (17.9%) | 28 ( 1.3%) |  |
| Q4 | 2,083 (74.7%) | 282 (12.1%) | 34 ( 1.4%) | 4 ( 0.2%) |  |
| Age (Years) | 2.00 (1.00-2.00) | 1.00 (1.00-2.00) | 1.00 (1.00-2.00) | 2.00 (1.00-2.00) | <0.001 |
| Sex |  |  |  |  | <0.001 |
| Male | 1,070 (38.4%) | 1,006 (43.2%) | 1,165 (49.0%) | 1,252 (57.7%) |  |
| Female | 1,720 (61.6%) | 1,322 (56.8%) | 1,212 (51.0%) | 917 (42.3%) |  |
| Education |  |  |  |  | <0.001 |
| Low | 582 (20.9%) | 356 (15.3%) | 332 (14.0%) | 317 (14.6%) |  |
| Medium | 824 (29.5%) | 688 (29.6%) | 655 (27.6%) | 573 (26.4%) |  |
| High | 1,383 (49.6%) | 1,284 (55.2%) | 1,389 (58.5%) | 1,279 (59.0%) |  |
| Smoking Status |  |  |  |  | <0.001 |
| Non-Smoker | 1,865 (69.2%) | 1,496 (66.3%) | 1,458 (62.7%) | 1,212 (57.1%) |  |
| Smoker | 422 (15.7%) | 422 (18.7%) | 454 (19.5%) | 530 (25.0%) |  |
| Ex-Smoker | 409 (15.2%) | 338 (15.0%) | 414 (17.8%) | 382 (18.0%) |  |
| Nationality |  |  |  |  | <0.001 |
| Non-Qatari | 330 (11.8%) | 278 (11.9%) | 309 (13.0%) | 339 (15.6%) |  |
| Qatari | 2,460 (88.2%) | 2,050 (88.1%) | 2,068 (87.0%) | 1,830 (84.4%) |  |
| Income |  |  |  |  | <0.001 |
| <20k | 1,108 (43.3%) | 851 (39.9%) | 798 (36.5%) | 750 (37.1%) |  |
| 20k-50k | 874 (34.1%) | 802 (37.6%) | 834 (38.1%) | 756 (37.4%) |  |
| >80k | 578 (22.6%) | 482 (22.6%) | 556 (25.4%) | 516 (25.5%) |  |
| Leisure time physical activity (MET hours/week) | 3.00 (0.00-21.00) | 6.00 (0.00-22.50) | 6.00 (0.00-22.50) | 7.50 (0.00-28.50) | <0.001 |
| Sleep Duration |  |  |  |  | 0.78 |
| ≥7 hours | 1,056 (37.8%) | 886 (38.1%) | 879 (37.0%) | 833 (38.4%) |  |
| <7 hours | 1,734 (62.2%) | 1,441 (61.9%) | 1,497 (63.0%) | 1,334 (61.6%) |  |
| Obesity |  |  |  |  | <0.001 |
| Lean | 1,359 (48.8%) | 1,294 (55.6%) | 1,373 (57.8%) | 1,337 (61.7%) |  |
| Obese | 1,428 (51.2%) | 1,034 (44.4%) | 1,003 (42.2%) | 831 (38.3%) |  |
| eGFR (mL/min/1.73 m2) | 142.99 (14.65) | 142.68 (13.02) | 141.01 (13.63) | 138.91 (14.42) | <0.001 |
| Supplements Use |  |  |  |  | <0.001 |
| No | 1,065 (38.2%) | 926 (39.8%) | 1,009 (42.4%) | 942 (43.4%) |  |
| Yes | 1,725 (61.8%) | 1,402 (60.2%) | 1,368 (57.6%) | 1,227 (56.6%) |  |
| Vitamin D (ng/ml) | 17.00 (12.00-24.00) | 16.00 (11.00-23.00) | 16.00 (11.00-23.00) | 16.00 (11.00-22.00) | <0.001 |
| Dietary Patterns |  |  |  |  |  |
| Traditional | -0.02 (0.99) | 0.05 (1.03) | 0.02 (0.98) | -0.05 (1.00) | 0.008 |
| Prudent | -0.00 (0.97) | -0.01 (1.02) | -0.00 (1.00) | 0.01 (1.01) | 0.94 |
| Sweets/Fast Food | 0.02 (1.01) | 0.01 (1.01) | -0.00 (0.96) | -0.04 (1.03) | 0.19 |
| Metabolic Parameters |  |  |  |  |  |
| Cholesterol Total (mmol/L) | 4.88 (0.96) | 4.98 (0.94) | 5.05 (0.97) | 5.14 (0.98) | <0.001 |
| HDL Cholesterol Total (mmol/L) | 1.35 (0.37) | 1.39 (0.38) | 1.38 (0.38) | 1.36 (0.39) | 0.001 |
| Triglyceride (mmol/L) | 1.20 (0.80-1.70) | 1.10 (0.80-1.60) | 1.10 (0.80-1.50) | 1.10 (0.80-1.60) | <0.001 |
| LDL Cholesterol Total (mmol/L) | 2.91 (0.86) | 3.01 (0.86) | 3.09 (0.89) | 3.19 (0.93) | <0.001 |
| Glucose (mmol/L) | 5.10 (4.60-6.30) | 5.00 (4.60-5.50) | 5.00 (4.60-5.40) | 5.00 (4.60-5.40) | <0.001 |
| Systolic Blood Pressure (mmHg) | 116.40 (15.31) | 113.71 (14.11) | 113.90 (14.42) | 114.31 (14.82) | <0.001 |
| Diastolic Blood Pressure (mmHg) | 67.39 (10.07) | 66.94 (9.86) | 67.25 (10.26) | 68.23 (10.45) | <0.001 |
| Waist Circumference | 91.19 (14.83) | 88.84 (14.09) | 88.79 (13.82) | 89.48 (13.51) | <0.001 |
| Diabetes |  |  |  |  | <0.001 |
| No | 1,901 (68.1%) | 1,909 (82.0%) | 2,092 (88.0%) | 1,944 (89.6%) |  |
| Yes | 889 (31.9%) | 419 (18.0%) | 285 (12.0%) | 225 (10.4%) |  |
| Dyslipidemia |  |  |  |  | 0.006 |
| No | 955 (34.2%) | 885 (38.0%) | 874 (36.8%) | 734 (33.8%) |  |
| Yes | 1,835 (65.8%) | 1,443 (62.0%) | 1,503 (63.2%) | 1,435 (66.2%) |  |
| Depression |  |  |  |  | 0.029 |
| No | 1,359 (48.7%) | 1,127 (48.4%) | 1,192 (50.1%) | 1,136 (52.4%) |  |
| Yes | 1,431 (51.3%) | 1,201 (51.6%) | 1,185 (49.9%) | 1,033 (47.6%) |  |

**S6** Sample Characteristics by Calcium Quartiles

| Ca Quartiles | Q1 | Q2 | Q3 | Q4 | p-value |
| --- | --- | --- | --- | --- | --- |
|  | N=2,663 | N=2,471 | N=2,239 | N=2,291 |  |
| Magnesium (mmol/L) | 0.83 (0.06) | 0.83 (0.06) | 0.83 (0.07) | 0.82 (0.07) | <0.001 |
| Quartiles of Magnesium |  |  |  |  | <0.001 |
| Q1 | 671 (25.2%) | 668 (27.0%) | 670 (29.9%) | 781 (34.1%) |  |
| Q2 | 654 (24.6%) | 581 (23.5%) | 553 (24.7%) | 540 (23.6%) |  |
| Q3 | 704 (26.4%) | 644 (26.1%) | 525 (23.4%) | 504 (22.0%) |  |
| Q4 | 634 (23.8%) | 578 (23.4%) | 491 (21.9%) | 466 (20.3%) |  |
| Calcium (mmol/L) | 2.21 (0.05) | 2.28 (0.01) | 2.33 (0.01) | 2.41 (0.05) | <0.001 |
| Metabolic Syndrome |  |  |  |  | <0.001 |
| No | 2,282 (85.8%) | 2,051 (83.1%) | 1,767 (79.1%) | 1,624 (71.0%) |  |
| Yes | 379 (14.2%) | 418 (16.9%) | 468 (20.9%) | 664 (29.0%) |  |
| Ca to Mg Ratio | 2.66 (0.21) | 2.76 (0.22) | 2.84 (0.24) | 2.97 (0.30) | <0.001 |
| Quartiles of Ca to Mg Ratio |  |  |  |  | <0.001 |
| Q1 | 1,201 (45.1%) | 680 (27.5%) | 377 (16.8%) | 173 ( 7.6%) |  |
| Q2 | 733 (27.5%) | 728 (29.5%) | 559 (25.0%) | 385 (16.8%) |  |
| Q3 | 492 (18.5%) | 609 (24.6%) | 692 (30.9%) | 632 (27.6%) |  |
| Q4 | 237 ( 8.9%) | 454 (18.4%) | 611 (27.3%) | 1,101 (48.1%) |  |
| Age (Years) | 1.00 (1.00-2.00) | 1.00 (1.00-2.00) | 1.00 (1.00-2.00) | 2.00 (1.00-2.00) | <0.001 |
| Sex |  |  |  |  | <0.001 |
| Male | 1,248 (46.9%) | 1,184 (47.9%) | 1,082 (48.3%) | 979 (42.7%) |  |
| Female | 1,415 (53.1%) | 1,287 (52.1%) | 1,157 (51.7%) | 1,312 (57.3%) |  |
| Education |  |  |  |  | <0.001 |
| Low | 326 (12.2%) | 333 (13.5%) | 377 (16.8%) | 551 (24.1%) |  |
| Medium | 757 (28.4%) | 707 (28.6%) | 655 (29.3%) | 621 (27.1%) |  |
| High | 1,579 (59.3%) | 1,431 (57.9%) | 1,206 (53.9%) | 1,119 (48.8%) |  |
| Smoking Status |  |  |  |  | 0.17 |
| Non-Smoker | 1,639 (63.6%) | 1,518 (63.4%) | 1,374 (63.0%) | 1,500 (66.8%) |  |
| Smoker | 507 (19.7%) | 478 (19.9%) | 437 (20.0%) | 406 (18.1%) |  |
| Ex-Smoker | 431 (16.7%) | 400 (16.7%) | 371 (17.0%) | 341 (15.2%) |  |
| Nationality |  |  |  |  | 0.46 |
| Non-Qatari | 343 (12.9%) | 302 (12.2%) | 308 (13.8%) | 303 (13.2%) |  |
| Qatari | 2,320 (87.1%) | 2,169 (87.8%) | 1,931 (86.2%) | 1,988 (86.8%) |  |
| Income |  |  |  |  | <0.001 |
| <20k | 913 (37.4%) | 861 (37.5%) | 807 (39.2%) | 926 (43.9%) |  |
| 20k-50k | 919 (37.7%) | 887 (38.6%) | 781 (37.9%) | 679 (32.2%) |  |
| >80k | 606 (24.9%) | 549 (23.9%) | 472 (22.9%) | 505 (23.9%) |  |
| Leisure time physical activity (MET hours/week) | 6.00 (0.00-26.00) | 6.50 (0.00-24.00) | 6.00 (0.00-22.88) | 3.00 (0.00-18.75) | <0.001 |
| Obesity |  |  |  |  | <0.001 |
| Lean | 1,610 (60.5%) | 1,363 (55.2%) | 1,266 (56.5%) | 1,124 (49.1%) |  |
| Obese | 1,052 (39.5%) | 1,107 (44.8%) | 973 (43.5%) | 1,164 (50.9%) |  |
| eGFR (mL/min/1.73 m2) | 142.97 (13.08) | 142.10 (13.67) | 141.36 (13.80) | 139.33 (15.48) | <0.001 |
| Vitamin D (ng/ml) | 15.00 (11.00-21.00) | 16.00 (11.00-22.00) | 17.00 (12.00-23.00) | 18.00 (13.00-25.00) | <0.001 |
| Dietary Patterns |  |  |  |  |  |
| Traditional | 0.07 (0.99) | 0.01 (0.98) | 0.01 (1.00) | -0.10 (1.02) | <0.001 |
| Prudent | -0.03 (1.00) | -0.03 (0.98) | 0.00 (1.00) | 0.06 (1.02) | 0.013 |
| Sweets/Fast Food | 0.02 (1.03) | 0.04 (1.01) | -0.02 (0.96) | -0.04 (0.99) | 0.019 |
| Metabolic Parameters |  |  |  |  |  |
| Cholesterol Total (mmol/L) | 4.87 (0.94) | 4.97 (0.91) | 5.02 (0.94) | 5.19 (1.06) | <0.001 |
| HDL Cholesterol Total (mmol/L) | 1.37 (0.38) | 1.38 (0.38) | 1.37 (0.38) | 1.36 (0.37) | 0.45 |
| Triglyceride (mmol/L) | 1.00 (0.70-1.40) | 1.10 (0.80-1.50) | 1.10 (0.80-1.60) | 1.20 (0.90-1.80) | <0.001 |
| LDL Cholesterol Total (mmol/L) | 2.96 (0.87) | 3.02 (0.85) | 3.05 (0.86) | 3.16 (0.96) | <0.001 |
| Glucose (mmol/L) | 4.90 (4.60-5.40) | 5.00 (4.60-5.50) | 5.00 (4.60-5.60) | 5.10 (4.60-6.10) | <0.001 |
| Systolic Blood Pressure (mmHg) | 112.29 (14.02) | 113.85 (14.40) | 114.91 (14.26) | 118.08 (15.71) | <0.001 |
| Diastolic Blood Pressure (mmHg) | 66.90 (10.27) | 67.40 (10.13) | 67.43 (10.07) | 68.09 (10.14) | <0.001 |
| Waist Circumference | 87.56 (13.60) | 89.47 (14.11) | 89.80 (13.90) | 92.13 (14.66) | <0.001 |
| Diabetes |  |  |  |  | <0.001 |
| No | 2,335 (87.7%) | 2,072 (83.9%) | 1,800 (80.4%) | 1,639 (71.5%) |  |
| Yes | 328 (12.3%) | 399 (16.1%) | 439 (19.6%) | 652 (28.5%) |  |
| Dyslipidemia |  |  |  |  | <0.001 |
| No | 1,099 (41.3%) | 944 (38.2%) | 772 (34.5%) | 633 (27.6%) |  |
| Yes | 1,564 (58.7%) | 1,527 (61.8%) | 1,467 (65.5%) | 1,658 (72.4%) |  |

**S7** Association between Serum Magnesium (Mg) Levels and Components of Metabolic Syndrome

| Outcome | Serum Mg Quartiles | Odds Ratio | P-value | 95% CI | P for Trend |
| --- | --- | --- | --- | --- | --- |
| Elevated Triglycerides^1^ |  |  |  |  | <0.001 |
|  | Q1 | **1.80** | **<0.001** | **(1.50 2.16)** |  |
|  | Q2 | **1.23** | **0.036** | **(1.01-1.51)** |  |
|  | Q3 | 1.02 | 0.796 | (0.83-1.26) |  |
|  | Q4 | 1.00 |  |  |  |
| Reduced HDL^2^ |  |  |  |  | <0.001 |
|  | Q1 | **1.45** | **<0.001** | **(1.28-1.64)** |  |
|  | Q2 | 1.13 | 0.055 | (0.99-1.29) |  |
|  | Q3 | 0.97 | 0.750 | (0.86-1.11) |  |
|  | Q4 | 1.00 |  |  |  |
| Elevated Glucose^3^ |  |  |  |  | <0.001 |
|  | Q1 | **2.39** | **<0.001** | **(2.08-2.76)** |  |
|  | Q2 | **1.44** | **<0.001** | **(1.23-1.67)** |  |
|  | Q3 | 1.08 | 0.294 | (0.93-1.26) |  |
|  | Q4 | 1.00 |  |  |  |
| Elevated Blood Pressure^4^ |  |  |  |  | <0.001 |
|  | Q1 | **1.31** | **<0.001** | **(1.14-1.52)** |  |
|  | Q2 | **0.84** | **0.040** | **(0.72-0.99)** |  |
|  | Q3 | 0.89 | 0.172 | (0.76-1.04) |  |
|  | Q4 | 1.00 |  |  |  |
| Central Obesity^5^ |  |  |  |  | <0.001 |
|  | Q1 | **1.41** | **<0.001** | **(1.24-1.61)** |  |
|  | Q2 | 1.11 | 0.117 | (0.97-1.28) |  |
|  | Q3 | 1.09 | 0.178 | (0.95-1.26) |  |
|  | Q4 | 1.00 |  |  |  |

(Q1 0.41-0.79, Q2 0.80-0.83, Q3 0.84-0.87, Q4 0.88-1.51). 1 Adjusted for age, obesity, smoking status, physical activity (n=9397); 2 Adjusted for age, sex, obesity, smoking status (n=9393);3 Adjusted for age, obesity, smoking status, physical activity (n=9398); 4. Adjusted for obesity, smoking status, dyslipidemia, physical activity (n=9395); 5. Adjusted for age, sex, smoking status, physical activity, diet, hyperglycemia (n=9399). Bold fonts represent statistically significant odds ratios (95% CI).

**S8** Association between Serum Calcium (Ca) Levels and Components of Metabolic Syndrome

| Outcome | Serum Ca Quartiles | Odds Ratio | P-value | 95% CI | P for Trend |
| --- | --- | --- | --- | --- | --- |
| Elevated Triglycerides^1^ |  |  |  |  | <0.001 |
|  | Q1 | 1.00 |  |  |  |
|  | Q2 | 1.31 | 0.007 | (1.07-1.61) |  |
|  | Q3 | **1.38** | **0.001** | **(1.13-1.69)** |  |
|  | Q4 | **1.72** | **<0.001** | **(1.42-2.08)** |  |
| Reduced HDL^2^ |  |  |  |  | 0.035 |
|  | Q1 | 1.00 |  |  |  |
|  | Q2 | 0.97 | 0.694 | (0.86-1.10) |  |
|  | Q3 | 1.06 | 0.300 | (0.94-1.20) |  |
|  | Q4 | 1.11 | 0.075 | (0.98-1.26) |  |
| Elevated Glucose^3^ |  |  |  |  | <0.001 |
|  | Q1 | 1.00 |  |  |  |
|  | Q2 | 1.11 | 0.124 | (0.96-1.28) |  |
|  | Q3 | **1.40** | **<0.001** | **(1.21-1.62)** |  |
|  | Q4 | **1.62** | **<0.001** | **(1.41-1.86)** |  |
| Elevated Blood Pressure^4^ |  |  |  |  | <0.001 |
|  | Q1 | 1.00 |  |  |  |
|  | Q2 | 1.03 | 0.682 | (0.87-1.21) |  |
|  | Q3 | **1.28** | **0.003** | **(1.08-1.50)** |  |
|  | Q4 | **1.71** | **<0.001** | **(1.46-1.99)** |  |
| Central Obesity^5^ |  |  |  |  | <0.001 |
|  | Q1 | 1.00 |  |  |  |
|  | Q2 | **1.27** | **<0.001** | **(1.11-1.44)** |  |
|  | Q3 | 1.20 | 0.006 | (1.05-1.38) |  |
|  | Q4 | **1.35** | **<0.001** | **(1.18-1.54)** |  |

(Q1 1.45-2.25, Q2 2.26-2.30, Q3 2.31-2.35, Q4 2.36-2.86). 1Adjusted for age, obesity, smoking status, vitamin D status (n=9334);2Adjusted for age, sex, obesity, smoking status, physical activity, vitamin D status (n=9330);3Adjusted for age, sex, obesity, vitamin D status, diet (n=9593);4Adjusted for age, obesity, smoking status, physical activity, dyslipidemia, diet (n=9395); 5Adjusted for age, sex, smoking status, physical activity, vitamin D status, diet, hyperglycemia (n=9336). Bold fonts represent statistically significant odds ratios (95% CI).

**S9** Subgroup Analysis of the Association Between Serum Ca:Mg Ratio and MetS

|  | **Quartiles of Ca to Mg Ratio** | | | |  |  |
| --- | --- | --- | --- | --- | --- | --- |
|  | **Q1** | **Q2** | **Q3** | **Q4** | **p for trend** | **p for interaction** |
| **Age (Years)** |  |  |  |  |  | 0.850 |
| Young | 1.00 | 1.21 (0.79-1.83) | 1.68 (1.13-2.48) | 2.66 (1.81-3.92) | <0.001 |  |
| Old | 1.00 | 1.17 (0.97-1.42) | 1.55 (1.29-1.88) | 2.78 (2.33-3.32) | <0.001 |  |
| **Sex** |  |  |  |  |  | 0.253 |
| Male | 1.00 | 1.17 (0.93-1.48) | 1.52 (1.20-1.91) | 2.37 (1.89-2.97) | <0.001 |  |
| Female | 1.00 | 1.21 (0.93-1.58) | 1.68 (1.31-2.17) | 3.13 (2.47-3.95) | <0.001 |  |
| **Education** |  |  |  |  |  | 0.347 |
| Low | 1.00 | 1.55 (1.08-2.23) | 1.69 (1.18-2.42) | 3.27 (2.36-4.53) | <0.001 |  |
| Medium | 1.00 | 1.10 (0.75-1.62) | 1.89 (1.32-2.70) | 2.69 (1.92-3.77) | <0.001 |  |
| High | 1.00 | 1.11 (0.88-1.41) | 1.45 (1.15-1.83) | 2.60 (2.08-3.26) | <0.001 |  |
| **Smoking Status** |  |  |  |  |  | 0.447 |
| Non-Smoker | 1.00 | 1.19 (0.96-1.49) | 1.51 (1.22-1.87) | 2.82 (2.31-3.45) | <0.001 |  |
| Smoker | 1.00 | 0.91 (0.62-1.35) | 1.49 (1.02-2.18) | 2.14 (1.47-3.12) | <0.001 |  |
| Ex-Smoker | 1.00 | 1.57 (1.00-2.45) | 2.17 (1.40-3.37) | 3.31 (2.17-5.05) | <0.001 |  |
| **Leisure time PA (MET hours/week)** |  |  |  |  |  | 0.412 |
| T1 | 1.00 | 1.23 (0.95-1.60) | 1.40 (1.08-1.80) | 2.68 (2.12-3.39) | <0.001 |  |
| T2 | 1.00 | 1.17 (0.84-1.63) | 2.01 (1.46-2.76) | 3.18 (2.34-4.32) | <0.001 |  |
| T3 | 1.00 | 1.12 (0.79-1.57) | 1.55 (1.11-2.16) | 2.47 (1.78-3.42) | <0.001 |  |
| **Vitamin D Status** |  |  |  |  |  | 0.394 |
| Adequacy | 1.00 | 1.38 (1.05-1.82) | 1.85 (1.42-2.42) | 3.34 (2.60-4.29) | <0.001 |  |
| Inadequacy | 1.00 | 1.13 (0.85-1.50) | 1.41 (1.06-1.87) | 2.65 (2.03-3.46) | <0.001 |  |
| Deficiency | 1.00 | 0.94 (0.64-1.39) | 1.37 (0.95-1.98) | 1.79 (1.24-2.59) | <0.001 |  |
| **Supplement Use** |  |  |  |  |  | **0.192** |
| No | 1.00 | 0.98 (0.74-1.28) | 1.31 (1.00-1.72) | **2.56 (1.98-3.31)** | <0.001 |  |
| Yes | 1.00 | 1.37 (1.09-1.73) | 1.78 (1.43-2.21) | **2.85 (2.31-3.51)** | <0.001 |  |
| **Sleep Duration** |  |  |  |  |  | 0.436 |
| ≥7 hours | 1.00 | 1.40 (1.04-1.88) | 1.62 (1.21-2.16) | 2.80 (2.13-3.69) | <0.001 |  |
| <7 hours | 1.00 | 1.07 (0.86-1.33) | 1.55 (1.26-1.92) | 2.70 (2.21-3.30) | <0.001 |  |
| **Depression** |  |  |  |  |  | 0.695 |
| No | 1.00 | 1.22 (0.97-1.53) | 1.57 (1.25-1.96) | 2.90 (2.34-3.58) | <0.001 |  |
| Yes | 1.00 | 1.12 (0.86-1.48) | 1.60 (1.24-2.08) | 2.57 (2.00-3.30) | <0.001 |  |

**S10** Subgroup Analysis of the Association Between Serum Mg and MetS

|  | Quartiles of Magnesium | | | |  |  |
| --- | --- | --- | --- | --- | --- | --- |
|  | Q1 | Q2 | Q3 | Q4 | p for trend | p for interaction |
| Age (Years) |  |  |  |  |  | 0.875 |
| Young | 2.48 (1.67-3.70) | 1.61 (1.06-2.45) | 1.19 (0.77-1.84) | 1.00 | <0.001 |  |
| Old | 2.43 (2.04-2.89) | 1.37 (1.13-1.66) | 1.10 (0.91-1.34) | 1.00 | <0.001 |  |
| Sex |  |  |  |  |  | 0.999 |
| Male | 2.40 (1.93-3.00) | 1.39 (1.09-1.76) | 1.12 (0.88-1.42) | 1.00 | <0.001 |  |
| Female | 2.42 (1.91-3.06) | 1.43 (1.11-1.85) | 1.12 (0.86-1.45) | 1.00 | <0.001 |  |
| Education |  |  |  |  |  | 0.161 |
| Low | 2.61 (1.90-3.59) | 1.28 (0.90-1.81) | 1.16 (0.81-1.66) | 1.00 | <0.001 |  |
| Medium | 2.60 (1.83-3.70) | 2.12 (1.46-3.08) | 1.25 (0.84-1.85) | 1.00 | <0.001 |  |
| High | 2.30 (1.84-2.87) | 1.24 (0.98-1.58) | 1.07 (0.85-1.36) | 1.00 | <0.001 |  |
| Smoking Status |  |  |  |  |  | 0.868 |
| Non-Smoker | 2.29 (1.87-2.79) | 1.32 (1.06-1.63) | 1.09 (0.88-1.36) | 1.00 | <0.001 |  |
| Smoker | 2.67 (1.85-3.88) | 1.69 (1.14-2.51) | 1.23 (0.82-1.85) | 1.00 | <0.001 |  |
| Ex-Smoker | 2.66 (1.79-3.96) | 1.49 (0.95-2.34) | 1.01 (0.65-1.59) | 1.00 | <0.001 |  |
| Leisure time PA (MET hours/week) |  |  |  |  |  | 0.730 |
| T1 | 2.12 (1.68-2.68) | 1.19 (0.92-1.55) | 1.04 (0.80-1.35) | 1.00 | <0.001 |  |
| T2 | 2.89 (2.13-3.92) | 1.67 (1.21-2.32) | 1.25 (0.89-1.74) | 1.00 | <0.001 |  |
| T3 | 2.51 (1.83-3.46) | 1.59 (1.13-2.24) | 1.08 (0.76-1.55) | 1.00 | <0.001 |  |
| Vitamin D |  |  |  |  |  | 0.906 |
| Adequacy | 2.65 (2.08-3.37) | 1.49 (1.15-1.94) | 1.11 (0.85-1.45) | 1.00 | <0.001 |  |
| Inadequacy | 2.51 (1.91-3.30) | 1.50 (1.12-2.01) | 1.16 (0.86-1.55) | 1.00 | <0.001 |  |
| Deficiency | 1.75 (1.22-2.51) | 1.16 (0.78-1.71) | 0.99 (0.67-1.47) | 1.00 | 0.001 |  |
| Supplements Use |  |  |  |  |  | 0.291 |
| No | 2.25 (1.75-2.90) | 1.22 (0.93-1.61) | 0.93 (0.70-1.23) | 1.00 | <0.001 |  |
| Yes | 2.50 (2.03-3.08) | 1.56 (1.25-1.96) | 1.27 (1.01-1.60) | 1.00 | <0.001 |  |
| Sleep Duration |  |  |  |  |  | 0.572 |
| ≥7 hours | 2.54 (1.94-3.33) | 1.39 (1.03-1.86) | 1.28 (0.95-1.73) | 1.00 | <0.001 |  |
| <7 hours | 2.33 (1.91-2.84) | 1.41 (1.13-1.75) | 1.03 (0.83-1.28) | 1.00 | <0.001 |  |
| Depression |  |  |  |  |  | 0.980 |
| No | 2.44 (1.98-3.01) | 1.40 (1.12-1.76) | 1.10 (0.88-1.39) | 1.00 | <0.001 |  |
| Yes | 2.41 (1.88-3.10) | 1.45 (1.10-1.90) | 1.16 (0.88-1.53) | 1.00 | <0.001 |  |

**S11** Subgroup Analysis of the Association Between Serum Ca Ratio and MetS

|  | Quartiles of Calcium | | | |  |  |
| --- | --- | --- | --- | --- | --- | --- |
|  | Q1 | Q2 | Q3 | Q4 | p for trend | p for interaction |
| Age (Years) |  |  |  |  |  | 0.210 |
| Young | 1.00 | 1.18 (0.82-1.70) | 1.52 (1.06-2.17) | 1.30 (0.88-1.91) | 0.068 |  |
| Old | 1.00 | 1.16 (0.97-1.41) | 1.37 (1.13-1.64) | 1.79 (1.50-2.14) | <0.001 |  |
| Sex |  |  |  |  |  | 0.004 |
| Male | 1.00 | 1.04 (0.83-1.30) | 1.19 (0.95-1.50) | 1.29 (1.03-1.62) | 0.014 |  |
| Female | 1.00 | 1.33 (1.04-1.70) | 1.66 (1.30-2.11) | 2.24 (1.79-2.81) | <0.001 |  |
| Education |  |  |  |  |  | 0.238 |
| Low | 1.00 | 1.39 (0.97-1.99) | 1.68 (1.18-2.38) | 2.13 (1.54-2.94) | <0.001 |  |
| Medium | 1.00 | 0.97 (0.69-1.36) | 1.49 (1.07-2.08) | 1.54 (1.11-2.13) | 0.001 |  |
| High | 1.00 | 1.18 (0.94-1.48) | 1.25 (0.99-1.57) | 1.61 (1.29-2.01) | <0.001 |  |
| Smoking Status |  |  |  |  |  | 0.029 |
| Non-Smoker | 1.00 | 1.28 (1.04-1.58) | 1.59 (1.29-1.96) | 2.06 (1.70-2.51) | <0.001 |  |
| Smoker | 1.00 | 0.97 (0.66-1.42) | 1.25 (0.86-1.83) | 1.22 (0.83-1.79) | 0.166 |  |
| Ex-Smoker | 1.00 | 1.02 (0.68-1.53) | 0.93 (0.61-1.41) | 1.18 (0.79-1.76) | 0.536 |  |
| Leisure time PA (MET hours/week) |  |  |  |  |  | 0.224 |
| T1 | 1.00 | 1.24 (0.96-1.60) | 1.58 (1.23-2.02) | 2.03 (1.61-2.56) | <0.001 |  |
| T2 | 1.00 | 1.01 (0.74-1.37) | 1.34 (0.99-1.81) | 1.55 (1.16-2.08) | <0.001 |  |
| T3 | 1.00 | 1.23 (0.90-1.70) | 1.18 (0.84-1.65) | 1.41 (1.02-1.95) | 0.058 |  |
| Vitamin D |  |  |  |  |  | 0.740 |
| Adequacy | 1.00 | 1.37 (1.05-1.78) | 1.40 (1.08-1.82) | 1.81 (1.42-2.31) | <0.001 |  |
| Inadequacy | 1.00 | 1.06 (0.81-1.40) | 1.43 (1.09-1.87) | 1.62 (1.24-2.10) | <0.001 |  |
| Deficiency | 1.00 | 1.02 (0.71-1.47) | 1.32 (0.91-1.91) | 1.58 (1.09-2.29) | 0.007 |  |
| Supplements Use |  |  |  |  |  | 0.089 |
| No | 1.00 | 1.11 (0.85-1.46) | 1.70 (1.31-2.21) | 1.77 (1.37-2.29) | <0.001 |  |
| Yes | 1.00 | 1.19 (0.96-1.48) | 1.22 (0.98-1.51) | 1.68 (1.37-2.05) | <0.001 |  |
| Sleep Duration |  |  |  |  |  | 0.802 |
| ≥7 hours | 1.00 | 1.09 (0.82-1.44) | 1.29 (0.98-1.69) | 1.63 (1.25-2.12) | <0.001 |  |
| <7 hours | 1.00 | 1.22 (0.99-1.50) | 1.49 (1.21-1.83) | 1.80 (1.47-2.19) | <0.001 |  |
| Depression |  |  |  |  |  | 0.093 |
| No | 1.00 | 1.24 (0.99-1.54) | 1.40 (1.12-1.75) | 1.96 (1.58-2.43) | <0.001 |  |
| Yes | 1.00 | 1.11 (0.86-1.43) | 1.43 (1.12-1.84) | 1.47 (1.16-1.87) | <0.001 |  |
